# Control of Photoinduced Electron Transfer Using Complex Formation of Water-Soluble Porphyrin and Polyvinylpyrrolidone

**DOI:** 10.3390/polym14061191

**Published:** 2022-03-16

**Authors:** Yilin Cao, Tomoe Takasaki, Satoshi Yamashita, Yasuhisa Mizutani, Akira Harada, Hiroyasu Yamaguchi

**Affiliations:** 1 Department of Macromolecular Science, Graduate School of Science, Osaka University, 1-1 Machikaneyama, Toyonaka 560-0043, Osaka, Japan; caoy17@chem.sci.osaka-u.ac.jp (Y.C.); tomoe.takasaki@kuraray.com (T.T.); 2 Department of Chemistry, Graduate School of Science, Osaka University, 1-1 Machikaneyama, Toyonaka 560-0043, Osaka, Japan; yamashitas16@chem.sci.osaka-u.ac.jp; 3 The Institute of Scientific and Industrial Research, Osaka University, 8-1 Mihogaoka, Ibaraki 567-0047, Osaka, Japan; 4 Graduate School of Science and Project Research Center for Fundamental Sciences, Osaka University, 1-1 Machikaneyama, Toyonaka 560-0043, Osaka, Japan; 5 Innovative Catalysis Science Division, Institute for Open and Transdisciplinary Research Initiatives (OTRI), Osaka University, Suita 565-0871, Osaka, Japan

**Keywords:** photoinduced electron transfer, water-soluble porphyrin, polyvinylpyrrolidone, complex formation

## Abstract

Inspired by the natural photosynthetic system in which proteins control the electron transfer from electron donors to acceptors, in this research, artificial polymers were tried to achieve this control effect. Polyvinylpyrrolidone (PVP) was found to form complex with pigments 5,10,15,20-tetrakis-(4-sulfonatophenyl) porphyrin (TPPS) and its zinc complex (ZnTPPS) quantitatively through different interactions (hydrogen bonds and coordination bonds, respectively). These complex formations hinder the interaction between ground-state TPPS or ZnTPPS and an electron acceptor (methyl viologen, MV^2+^) and could control the photoinduced electron transfer from TPPS or ZnTPPS to MV^2+^, giving more electron transfer products methyl viologen cationic radical (MV^+•^). Other polymers such as PEG did not show similar results, indicating that PVP plays an important role in controlling the photoinduced electron transfer.

## 1. Introduction

With the depletion of fossil fuel resources and the increasingly severe environmental problems, the development and utilization of green energy such as solar energy, wind energy, water energy, and biomass energy have become more and more critical [1,2]. Among them, solar energy is the most abundant and continuously supplied energy on earth [3]. The best use of solar energy exists in nature. Natural photosynthetic systems can transform solar energy into chemical energy with high efficiency [4]. The pigments in natural photosynthetic reaction centers are fixed by the protein around them, making the distance and relative position of the electron donors and electron acceptors noncovalently fixed at the optimum conditions for electron transfer [4,5,6,7,8,9].

Extensive effort has been devoted to mimicking the natural photosynthetic systems to study the initial process of photosynthesis and to realize highly efficient electron transfer in artificial photosynthetic systems [10,11,12,13,14,15,16,17,18,19,20,21,22,23,24,25,26,27]. Numerous studies of electron transfer systems using covalently linked electron donor and acceptor molecules have been reported [10,11,12,13]. Recent covalently linked donor-acceptor systems are reported and used as polymerization photoinitiators [14,15]. Systems with noncovalently assembled electron donor and acceptor molecules have also been constructed via hydrogen bonding [16,17,18], metal coordination [18,19,20], electrostatic interaction [21,22,23], and host–guest interaction [24,25,26,27]. Among these studies, those using polymer matrix have attracted widespread interest because the polymer matrix can fix the electron donors, which is similar to a natural photosynthetic system. Takuzo Aida reported the use of porphyrin dendrimer to control the distance and electron transfer between porphyrin and methyl viologen [21]. Recently, Linqi Shi reported controlled electron transfer between porphyrin and water-soluble fullerene in poly(ethylene glycol)-block-poly(l-lysine) micelles [23]. However, the molecular design and synthesis are still complex. A convenient and generalized method is yet to be developed.

**Figure 1:**
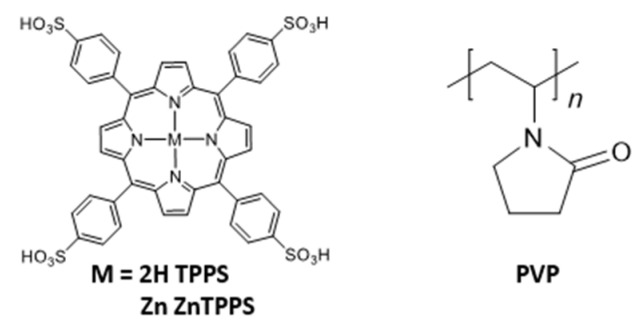
Chemical structures of 5,10,15,20-tetrakis-(4-sulfonatophenyl) porphyrin (TPPS), its zinc complex (ZnTPPS), and polyvinylpyrrolidone (PVP).

In the present work, polyvinylpyrrolidone (PVP, Figure 1, PVP refers to PVP K-30 (M_η_ = 40,000, M_η_ represents for the viscosity-average molecular weight) unless otherwise noted) as a synthetic polymer was found to form complexes with 5,10,15,20-tetrakis-(4-sulfonatophenyl) porphyrin (TPPS, Figure 1) and its zinc complex (ZnTPPS, Figure 1) through different interactions. We report here for the first time that the interaction between porphyrins and the electron acceptor, methyl viologen (MV^2+^) can be regulated in the presence of PVP, resulting in an increase in the concentration of electron-transfer products.

## 2. Materials and Methods

### 2.1. Materials

Tetraphenylporphyrin tetrasulfonic acid hydrate (TPPS) and polyethylene glycol 6000 (PEG 6000) were purchased from TCI Co., Ltd., Tokyo, Japan. Polyvinylpyrrolidone (PVP 25, K-30 and K-90), methyl viologen hydrate, potassium dihydrogenphosphate, dipotassium hydrogenphosphate and ethylenediamine-*N*,*N*,*N*′,*N*′-tetraacetic acid tetrasodium salt (EDTA) were purchased from Nacalai Tesque, Kyoto, Japan. All the reagents and solvents were used as received without further purification. Zinc *meso*-5,10,15,20-tetrakis-(4-sulfonatophenyl)porphyrin (ZnTPPS) was prepared according to the method reported by Flamigni et al. [28].

### 2.2. Methods

The ^1^H NMR spectra were obtained using a JEOL (Tokyo, Japan) JNM-ECA 500 MHz NMR spectrometer. Chemical shifts were referenced to sodium 3-(trimethylsilyl)-1-propanesulfonate (*δ* = 0.00 ppm) and the solvent value (*δ* = 4.79 ppm for D_2_O). UV–vis spectra were recorded on a SHIMADZU (Kyoto, Japan) UV-2500PC spectrophotometer at 25 °C using a cell with a 1 cm path length. Fluorescence spectra were recorded on a HITACHI F-2500 fluorescence spectrophotometer (Tokyo, Japan). Resonance Raman scattering of the ZnTPPS in the absence and presence of PVP or pyridine was excited by ~20 ns laser pulses of 425 nm generated using the second harmonic of a Ti:sapphire laser pumped by a Q-switched diode-pumped Nd-doped yttrium lithium fluoride (Nd:YLF) laser (TU-L, Photonics Industries, Ronkonkoma, NY, USA) at 1 kHz. The pulse energy at the sample was 0.5 μJ. The sample solution was placed in a glass tube used as a spinning cell, and the scattered Raman light was collected and focused onto the entrance slit of a spectrograph (iHR550, HORIBA Jobin Ybon, Kyoto, Japan) equipped with a charge-coupled-device (CCD) camera (SPEC-10:400B/LN-SN-U, Roper Scientific, Sarasota, FL, USA). The accumulation times for obtaining each spectrum were 5 min. The Raman shifts were calibrated using the Raman bands of cyclohexane. The calibration error was within 1 cm^−1^ for prominent bands. Irradiation experiments were carried out using a UV irradiation unit (SP-11, USHIO, Tokyo, Japan) equipped with a ND10 filter (HOYA, Tokyo, Japan).

## 3. Results and Discussion

### 3.1. Complex Formations

**Figure 2:**
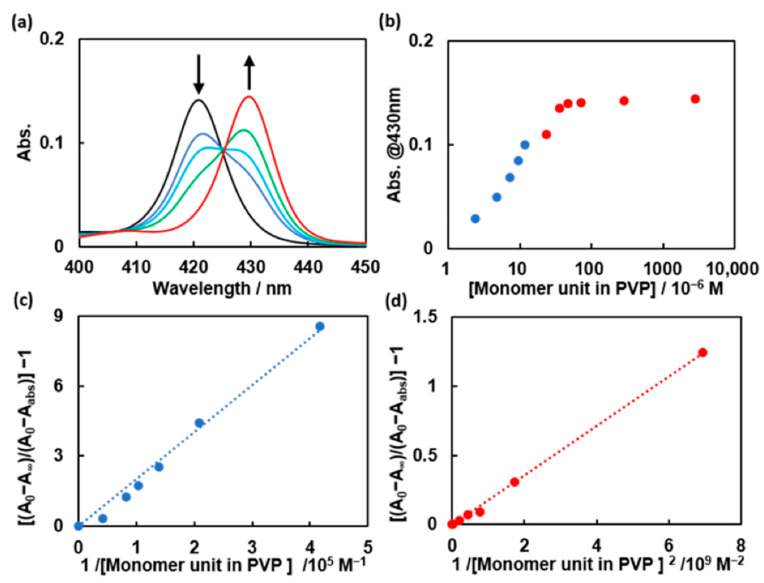
(**a**) Absorption spectra of 0.6 μM ZnTPPS upon the successive addition of 0, 4.8 μM, 7.2 μM, 12.0 μM and 2.9 mM PVP in 0.01 M phosphate buffer (pH = 8.0); (**b**) A plot of Abs. at 430 nm as a function of the concentration of PVP (the blue and red parts correspond to the different plot regions in (**c**,**d**); (**c**) Benesi–Hildebrand plots for the formation of ZnTPPS–PVP complex under the assumption of the formation of a 1:1 complex in the lower PVP concentration region; (**d**) Benesi–Hildebrand plots for the formation of ZnTPPS–PVP complex under the assumption of the formation of a 1:1 complex in the higher PVP concentration region.

We investigated the interaction between ZnTPPS and PVP by studying the absorption spectra of ZnTPPS in the absence and presence of PVP in 0.01 M phosphate buffer (pH = 8.0). Redshifts in the regions of the Soret band (Figure 2a and Figure S1) and the Q-bands (Figure S2) of ZnTPPS were observed upon the addition of PVP. These shifts suggest the formation of a complex between ZnTPPS and PVP.

Benesi–Hildebrand (BH) plots [29] were constructed, referring to the absorbance at 430 nm (Figure 2b), to determine the complex formation ratio and association constant of the ZnTPPS–PVP complex. BH plots corresponding to complex formation ratios of 1:1 and 1:2 in low and high PVP concentration regions are shown in Figure 2c,d, respectively. Both plots were linear in each PVP concentration region, suggesting that the ZnTPPS–PVP complex formed at a ratio of 1:1 (ZnTPPS:PVP_monomer unit_) and 1:2 in the lower and higher PVP concentration regions, respectively. The association constants were calculated from the slopes and were found to be *K*_1,Z_ = 5.0 × 10^4^ M^−1^ and *K*_1,Z_ × *K*_2,Z_ = 5.0 × 10^9^ M^−2^.

**Figure 3:**
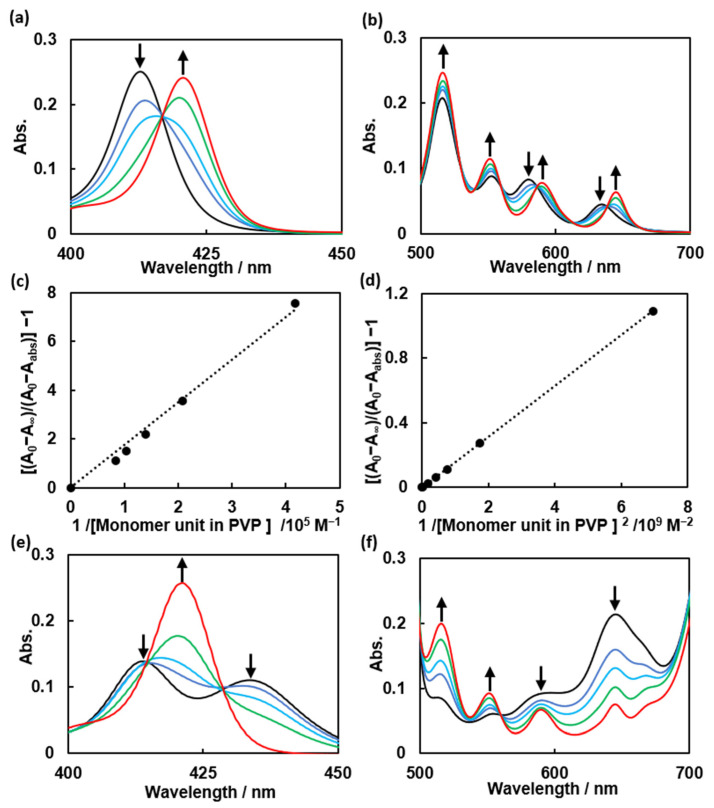
(**a**) Absorption spectra of 0.6 μM TPPS upon the successive addition of 0, 7.2 μM, 12.0 μM, 24.0 μM and 2.9 mM PVP in 0.01 M phosphate buffer (pH = 8.0); (**b**) absorption spectra of 12.0 μM TPPS upon the successive addition of 0, 0.14 mM, 0.24 mM, 0.48 mM and 0.06 M PVP in 0.01 M phosphate buffer (pH = 8.0); (**c**) Benesi–Hildebrand plots for the formation of a TPPS–PVP complex under the assumption of the formation of a 1:1 complex in the lower PVP concentration region; (**d**) Benesi–Hildebrand plots for the formation of a TPPS–PVP complex under the assumption of the formation of a 1:2 complex in the higher PVP concentration region. (**e**) Absorption spectra of 0.6 μM H_2_TPPS^2+^ upon the successive addition of 0, 7.2 μM, 12.0 μM, 24.0 μM and 2.9 mM PVP in 0.01 M phosphate buffer (pH = 4.0); (**f**) absorption spectra of 12.0 μM H_2_TPPS^2+^ upon the successive addition of 0, 0.14 mM, 0.24 mM or 0.48 mM and 0.06 M PVP in 0.01 M phosphate buffer (pH = 4.0).

The absorption spectra of TPPS in the presence and absence of PVP were also recorded in 0.01 M phosphate buffer (pH = 8.0). Redshifts in the Soret band and Q-bands were observed (Figure 3a,b, Figures S6 and S7), suggesting the formation of a complex between TPPS and PVP (TPPS–PVP complex). The corresponding BH plots were constructed in the same manner as those for the ZnTPPS–PVP complex (Figure 3c,d).The BH plots indicate that the TPPS–PVP complex formed at ratios of 1:1 (TPPS:PVP_monomer unit_) and 1:2 in the lower and higher PVP concentration regions, respectively. The association constants were calculated to be *K*_1,T_ = 5.0 × 10^4^ M^−1^ and *K*_1,T_ × *K*_2,T_ = 5.0 × 10^9^ M^−2^.

**Figure 4:**
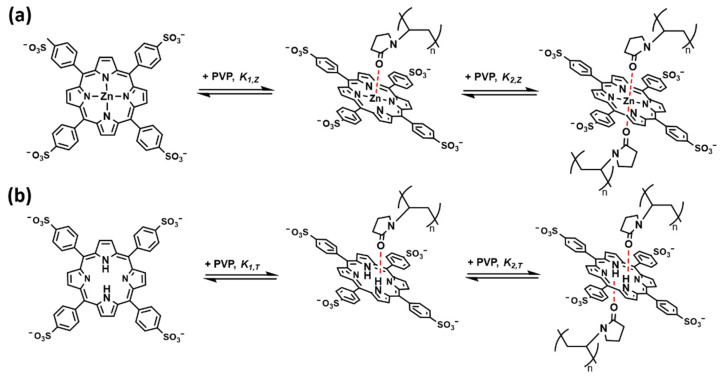
(**a**) Conceptual schemes of the formation of the ZnTPPS–PVP complex; (**b**) Conceptual schemes of the formation of the TPPS–PVP complex.

In addition to redshifts in the absorption spectra of ZnTPPS (12.0 μM), a color change (from purple to green) was also observed (Figure S3), both of which are phenomena observed when an agent interacts with zinc porphyrin via a coordination bond [30,31,32]. We speculated that a ZnTPPS–PVP complex forms through a coordination bond between the carbonyl group of PVP and the central Zn atom of ZnTPPS (Figure 4a); this interpretation is also supported by ^1^H NMR spectra (Figure S4) and Raman spectra (Figure S5).

Protonated TPPS (H_2_TPPS^2+^) was also examined at pH = 4.0. A Soret band of H_2_TPPS^2+^ appeared at 434 nm (Figure 3e and Figure S8), whereas the Q-bands appeared at 516 nm, 553 nm, 590 nm, and 645 nm (Figure 3f and Figure S9). The Q_y_ absorption bands became more intense than the Q_x_ bands. New bands at 490 nm and 708 nm, which corresponded to the formation of J-aggregates of TPPS [33], were also observed (Figure S9). Upon the addition of PVP, the Soret band H_2_TPPS^2+^ shifted to 430 nm. With a reversal of the absorption, the Q_y_ bands and Q_x_ bands shifted slightly, resulting in the Q-bands eventually assuming the same shape as that in the spectra corresponding to TPPS. We speculated that a TPPS–PVP complex formed via hydrogen bonding between the carbonyl group of PVP and the central amino group of TPPS (Figure 4b) and that the same complex formed under acidic pH conditions.

The fluorescence intensity of both TPPS and ZnTPPS (Figure S10) increased upon the addition of PVP, which suggests that the PVP inhibits the thermal fluctuation of porphyrins [25,27].

The absorption spectra of ZnTPPS or TPPS in the presence of *N*-methyl-2-pyrrolidone (NMP) (Figure S11) or *N*-vinyl-2-pyrrolidone (NVP) (Figure S11) indicate weak interactions between NMP or NVP and ZnTPPS or TPPS, suggesting that the polymer structure of PVP plays a critical role in the formation of the ZnTPPS–PVP and TPPS–PVP complexes. The interaction between ZnTPPS and PVP with different molecular weights (PVP 25 (M_η_ = 24,500), PVP K-90 (M_η_ = 360,000)) were also studied (Figure S12), and it showed no molecular weight dependence. Polyethylene glycol (PEG) was also used for testing, but PEG cannot form a complex with ZnTPPS (Figure S12).

### 3.2. Photoinduced Electron Transfer

**Figure 5:**
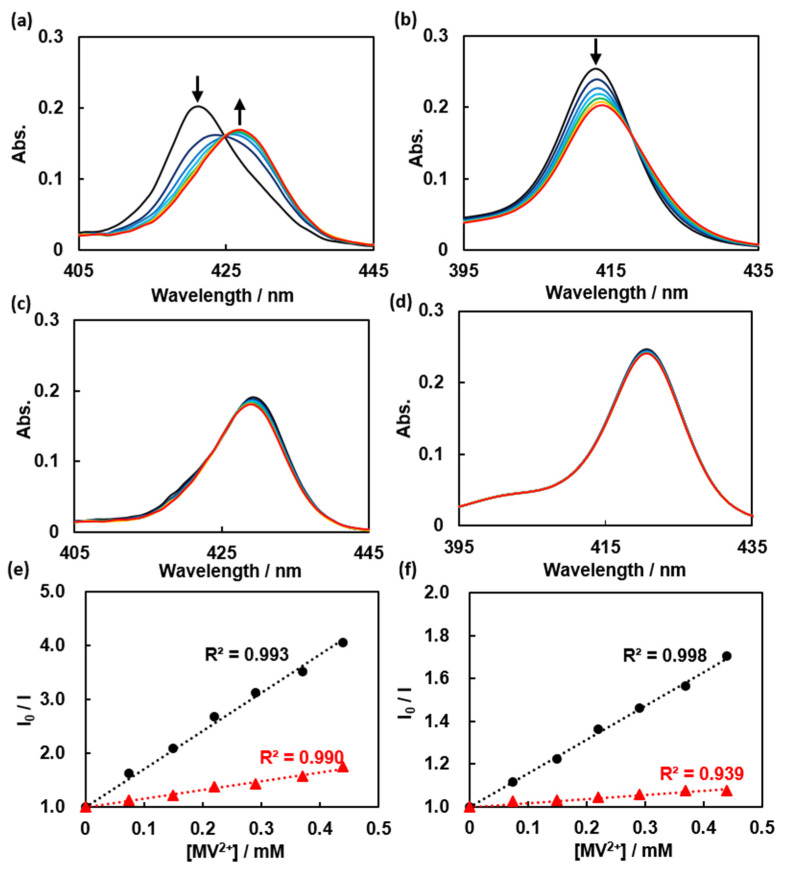
(**a**) Absorption spectra of 0.6 μM ZnTPPS with MV^2+^ added in 0.07 mM intervals up to 0.44 mM; (**b**) absorption spectra of 0.6 μM TPPS with MV^2+^ added in 0.07 mM intervals up to 0.44 mM; (**c**) absorption spectra of 0.6 μM ZnTPPS with MV^2+^ added in 0.07 mM intervals up to 0.44 mM in the presence of 24.0 μM PVP; (**d**) absorption spectra of 0.6 μM TPPS with MV^2+^ added in 0.07 mM intervals up to 0.44 mM in the presence of 24.0 μM PVP; (**e**) Stern–Volmer plots of the quenching of ZnTPPS by MV^2+^ in the absence (solid circles) and presence (solid triangles) of PVP; (**f**) Stern–Volmer plots of the quenching of TPPS by MV^2+^ in the absence (solid circles) and presence (solid triangles) of PVP.

In the presence of MV^2+^, ZnTPPS and TPPS can both form donor–acceptor pairs [34]. When MV^2+^ was added to TPPS or ZnTPPS, redshifts were observed in their absorption spectra (Figure 5a,b). After PVP was added to a TPPS or ZnTPPS solution, negligible peak shifts were observed upon the addition of MV^2+^ (Figure 5c,d). These results suggest that the ground-state interactions between the porphyrin and MV^2+^ were restrained. We observed the fluorescence quenching of ZnTPPS (Figure S13) and TPPS (Figure S14) by MV^2+^ in the presence and absence of PVP, excited at the isosbestic point, to investigate the electron transfer between the porphyrins and MV^2+^. Referring to the fluorescence intensity of spectra vertices Stern–Volmer (SV) plots for the quenching of the emission of the porphyrins were constructed (Figure 5e,f). Although the SV constant (*K*_sv_) decreased after the addition of PVP (*K*_SV, ZnTPPS–MV_: 7.1 to 1.6, *K*_SV, TPPS–MV_: 1.6 to 0.2), fluorescence quenching phenomena were actually observed.

No overlap exists in the emission spectra of porphyrin and absorption spectra of MV^2+^, so there is no Förster energy transfer. The fluorescence quenching experiments were carried out in the presence of oxygen, which could consume the triplet state porphyrin, and direct interaction between MV^2+^ and porphyrins was disturbed by PVP, making molecular collisions not likely to occur, so Dexter energy transfer could not be considered, which was also supported by the similar fluorescence quenching behavior in the presence and absence of oxygen (Figures S13b and S15a). The decrease in fluorescence intensity is attributed to photoinduced electron transfer at long distances.

PVP 25 and K-90 can also control the interaction and photoinduced electron transfer between ZnTPPS and MV^2+^, and the control effect of PVP showed no molecular weight dependence (Figures S16–S18). PEG can neither control the interaction nor the photoinduced electron transfer between ZnTPPS and MV^2+^ (Figures S16–S18).

**Figure 6:**
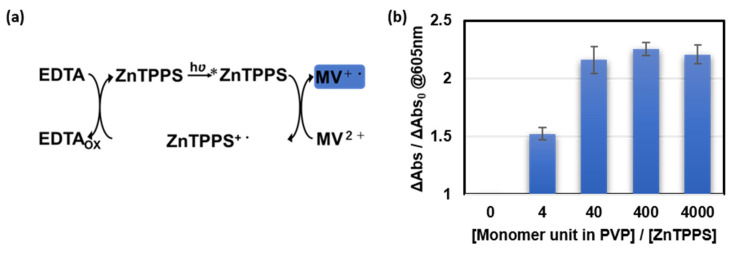
(**a**) Scheme showing the generation of MV^+•^ from MV^2+^ via electron transfer from photoexcited ZnTPPS (^*^ZnTPPS) with ethylenediamine-*N*,*N*,*N*′,*N*′-tetraacetic acid tetrasodium salt (EDTA) as a sacrificial agent; (**b**) Increase in the ratio of generated MV^+•^ in the presence of PVP compared with that in the polymer-free case.

The quantum yield of ZnTPPS is greater than that of TPPS [35] and the energy level of ZnTPPS is higher than TPPS [36], so electron transfer from ZnTPPS to MV^2+^ is easier to take place than that from TPPS, and ZnTPPS was used in subsequent experiments. Degassed solutions of ZnTPPS, PVP, MV^2+^, and a sacrificial agent (ethylenediamine-*N*,*N*,*N′*,*N′*-tetraacetic acid tetrasodium salt, EDTA) were irradiated with UV light at ~20 cm for 30 min, which changed the absorbance at 605 nm from a cationic radical of MV^2+^ (MV^+•^) [37] (ΔAbs) (Figure S19). The ratio of the generated amount of MV^+•^ compared with that in the polymer-free case (ΔAbs/ΔAbs_0_ at 605 nm) increased with increasing PVP concentration (Figure 6b) and saturated at high PVP concentrations. Even though the environment for electron transfer from ZnTPPS to MV^2+^ became burdened (i.e., the value of *K*_sv_ becomes much lower) after the addition of PVP, the ratio of the generated amount of MV^+•^ reached a maximum value of ~2.3. This result indicated that PVP plays a critical role in restricting the reverse electron transfer in this photoinduced electron-transfer process [24,26].

PVP 25 and K-90 can also give rise to the generated amount of MV^+•^ (Figures S20 and S21), and the generated amount of MV^+•^ showed little molecular weight dependence. PEG has no effects on the amount of MV^+•^ (Figures S20 and S21).

## 4. Conclusions

In the present work, PVP was found to form complexes with ZnTPPS and TPPS quantitatively. The interaction between PVP and ZnTPPS or TPPS was considered to be a coordination bond and hydrogen bond, respectively. The formation of ZnTPPS–PVP and TPPS–PVP complexes could control the interaction between ground-state porphyrins and MV^2+^ and the photoinduced electron transfer from porphyrins to MV^2+^. In the presence of PVP, more electron transfer products (MV^+•^) were generated through the photoinduced electron transfer from ZnTPPS to MV^2+^. The further transformation of the generated MV^+•^ to storable energy (e.g., hydrogen [27] or formic acid [38]) using catalysts to realize the conversion of solar energy to chemical energy is currently under investigation.

This study provides a convenient and generalized method to control photoinduced electron transfer between electron donor and acceptor. By using suitable polymers, we believe that this method can be applied to a wide range of electron donor and acceptor pairs.

## Data Availability

Not applicable.

## Supplementary Materials

The following supporting information can be downloaded at: https://www.mdpi.com/article/10.3390/polym14061191/s1, Figures S1–S21.
