# Supplementary material for: Control of Photoinduced Electron Transfer Using Complex Formation of Water-Soluble Porphyrin and Polyvinylpyrrolidone
Source: Polymers (Basel). 2022 Mar 16;14(6):1191. doi: 10.3390/polym14061191 (PMC8949476; doi:10.3390/polym14061191)
Supplement: Supplementary file 1 [file polymers-14-01191-s001.zip › polymers-1609301-supplementary.pdf]

Materials

Tetraphenylporphyrin tetrasulfonic acid (TPPS) and polyethylene glycol 6000 (PEG 6000) were purchased from TCI Co., Ltd. Polyvinylpyrrolidone (25, K-30, and K-90), methyl viologen hydrate, potassium dihydrogenphosphate, dipotassium hydrogenphosphate, and ethylenediamine-*N*,*N*,*N′*,*N′*-tetraacetic acid tetrasodium salt (EDTA-4Na salt) were purchased from Nacalai Tesque. All the reagents and solvents were used as received without further purification. Zinc *meso*-5,10,15,20-tetrakis-(4-sulfonatophenyl)porphyrin (ZnTPPS) was prepared according to the method reported by Flamigni et al. [1].

Measurements

The ^1H NMR spectra were obtained using a JEOL JNM-ECA 500 MHz NMR spectrometer. Chemical shifts were referenced to sodium 3-(trimethylsilyl)-1-propanesulfonate (δ = 0.00 ppm) and the solvent value (δ = 4.79 ppm for D_2O). UV–vis spectra were recorded on a SHIMADZU UV-2500PC spectrophotometer at 25 °C using a cell with a 1 cm path length. Fluorescence spectra were recorded on a HITACHI F-2500 fluorescence spectrophotometer. Resonance Raman scattering of the ZnTPPS in the absence and presence of PVP or pyridine was excited by ~20 ns laser pulses of 425 nm generated using the second harmonic of a Ti:sapphire laser pumped by a Q-switched diode-pumped Nd-doped yttrium lithium fluoride (Nd:YLF) laser (Photonics Industries, TU-L) at 1 kHz. The pulse energy at the sample was 0.5 μJ. The sample solution was placed in a glass tube used as a spinning cell, and the scattered Raman light was collected and focused onto the entrance slit of a spectrograph (HORIBA Jobin Ybon, iHR550) equipped with a charge-coupled-device (CCD) camera (Roper Scientific, SPEC-10:400B/LN-SN-U). The accumulation times for obtaining each spectrum were 5 min. The Raman shifts were calibrated using the Raman bands of cyclohexane. The calibration error was within 1 cm^{-1} for prominent bands. Irradiation experiments were carried out using a USHIO SP-11 UV irradiation unit equipped with a HOYA ND10 filter.

Absorption spectra of ZnTPPS upon the addition of PVP

Absorption spectra were acquired for mixed solutions of ZnTPPS (0.6 μM) and PVP (0, 2.4 μM, 4.8 μM, 7.2 μM, 9.6 μM, 12.0 μM, 24.0 μM, 36.0 μM, 48.0 μM, 72.0 μM, 0.3 mM, and 2.9 mM) in 0.01 M phosphate buffer (pH 8.0) (Figure S1). Redshifts in the regions of the Soret band and the Q-bands were observed. The accuracy of the Q-bands was not good due to the low concentration of ZnTPPS, therefore, absorption spectra were detected again in 20 folds the concentrations of ZnTPPS and PVP.

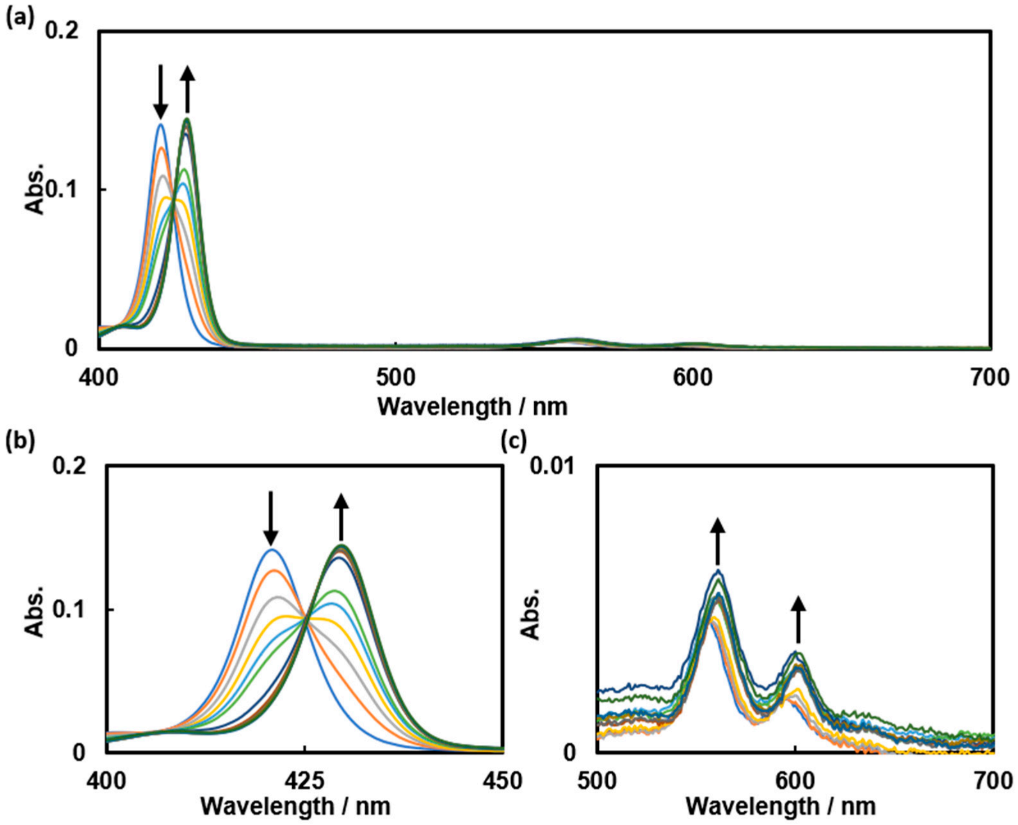

Figure S1. (a) Absorption spectra of 0.6 μM ZnTPPS upon the addition of 0, 2.4 μM, 4.8 μM, 7.2 μM, 9.6 μM, 12 μM, 24 μM, 36 μM, 48 μM, 72 μM, 0.29 mM, and 2.9 mM PVP in 0.01 M phosphate buffer (pH 8.0); (b) zoomed spectra of 400–500 nm of Figure S1a; (c) zoomed spectra of 500–700 nm of figure S1a.

Absorption spectra were acquired for mixed solutions of ZnTPPS (12 μM) and PVP (0, 0.05 mM, 0.10 mM, 0.14 mM, 0.19 mM, 0.24 mM, 0.48 mM, 0.72 mM, 0.96 mM, 1.44 mM, 5.76 mM, and 0.06 M) in 0.01 M phosphate buffer (pH 8.0) (Figure S2). Redshifts in the regions of the Soret band and the Q-bands were observed. The accuracy of the Soret band was not good due to the high concentration of ZnTPPS.

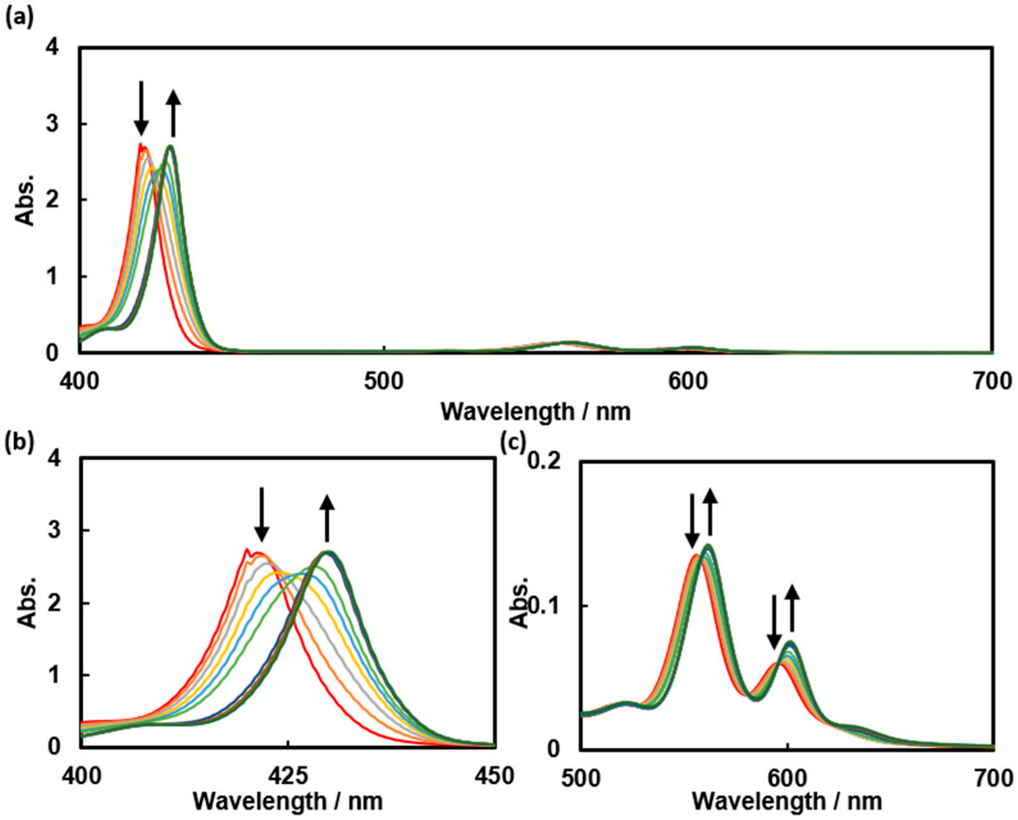

Figure S2. (a) Absorption spectra of 12 μM ZnTPPS upon the addition of 0, 0.05 mM, 0.10 mM, 0.14 mM, 0.19 mM, 0.24 mM, 0.48 mM, 0.72 mM, 0.96 mM, 1.44 mM, 5.76 mM, and 0.06 M PVP in 0.01 M phosphate buffer (pH 8.0); (b) zoomed spectra of 400–500 nm of Figure S2a; (c) zoomed spectra of 500–700 nm of Figure S2a.

Color change of ZnTPPS solution upon addition of PVP

The color of a 12 μM ZnTPPS solution changed from purple to green upon the addition of 0.06 mM PVP (Figure S3).

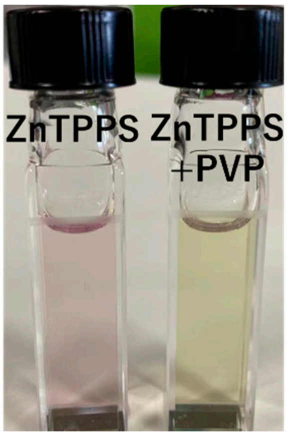

Figure S3. Color change of a 12 μM ZnTPPS solution upon the addition of 0.06 mM PVP.

^{1}H NMR spectra of ZnTPPS in the absence and presence of PVP

^{1}H NMR analyses of mixed solutions of ZnTPPS (10 mM) and PVP (0–100 mM, 0–10 equiv.) were carried out in D_2O at room temperature. The chemical shifts of the protons on the pyrrole rings exhibited the largest shifts (Figure S4), suggesting that PVP interacts with ZnTPPS in the center of a porphyrin ring.

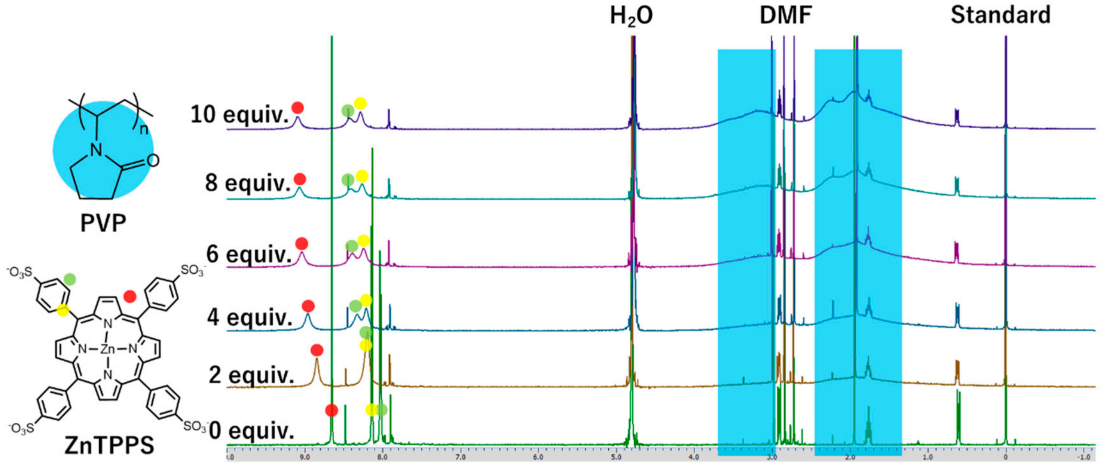

Figure S4. ^{1}H NMR spectra of 10 mM ZnTPPS in the absence and presence of 0–100 mM PVP, in D_2O at 25 °C.

Resonance Raman spectra of ZnTPPS in the absence and presence of PVP or pyridine

Resonance Raman spectra of mixed aqueous solutions of ZnTPPS (50 μM) in the absence and presence of PVP (4.5 mM, 90 equiv.) or pyridine (known to form complexes with ZnTPPS,[2] 4.5 mM, 90 equiv.) were acquired. The Raman band near 1450 cm^{-1} showed a 3 cm^{-1} downshift in the presence of PVP, whereas no shifts were observed upon the addition of pyridine (Figure S5). The frequency of this band is sensitive to the spin state (or coordination number) of the porphyrin.[3] The observed downshift indicated that the high-spin character of the porphyrin increased in the presence of PVP. We speculated that PVP interacts with ZnTPPS through a coordination bond between the carbonyl groups of PVP and the central zinc atom of ZnTPPS.

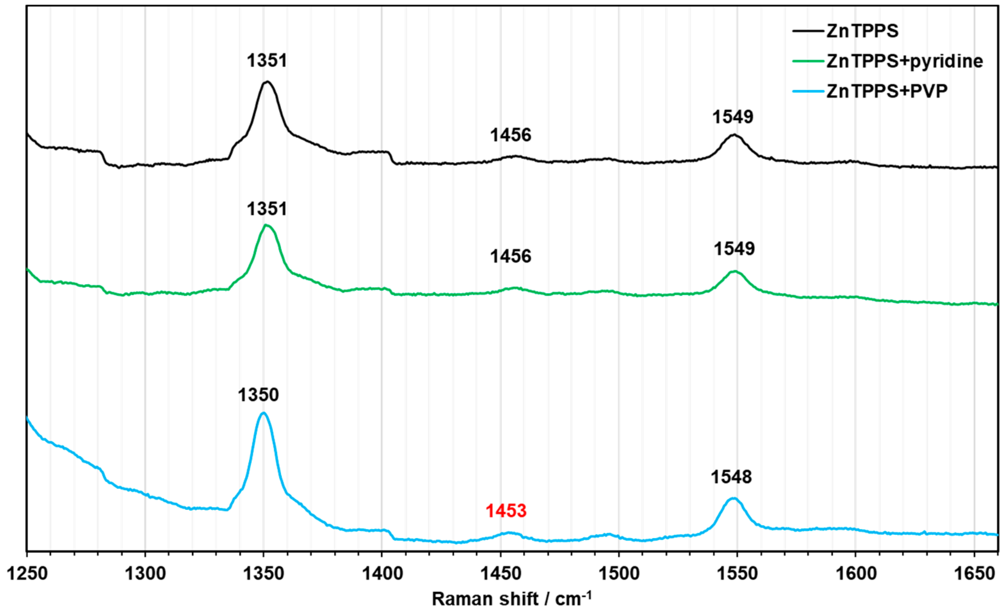

Figure S5. Raman spectra of 50 μM ZnTPPS in the absence and presence of 4.5 mM PVP or 4.5 mM pyridine.

Absorption spectra of TPPS upon the addition of PVP in 0.01 M phosphate buffer (pH 8.0)

Absorption spectra were acquired for mixed solutions of TPPS (0.6 μM) and PVP (0, 2.4 μM, 4.8 μM, 7.2 μM, 9.6 μM, 12.0 μM, 24.0 μM, 36.0 μM, 48.0 μM, 72.0 μM, 0.3 mM, and 2.9 mM) in 0.01 M phosphate buffer (pH 8.0) (Figure S6). Redshifts in the regions of the Soret band and the Q-bands were observed. The accuracy of the Q-bands was not good due to the low concentration of TPPS. So, absorption spectra were detected again in 20 folds the concentrations of TPPS and PVP.

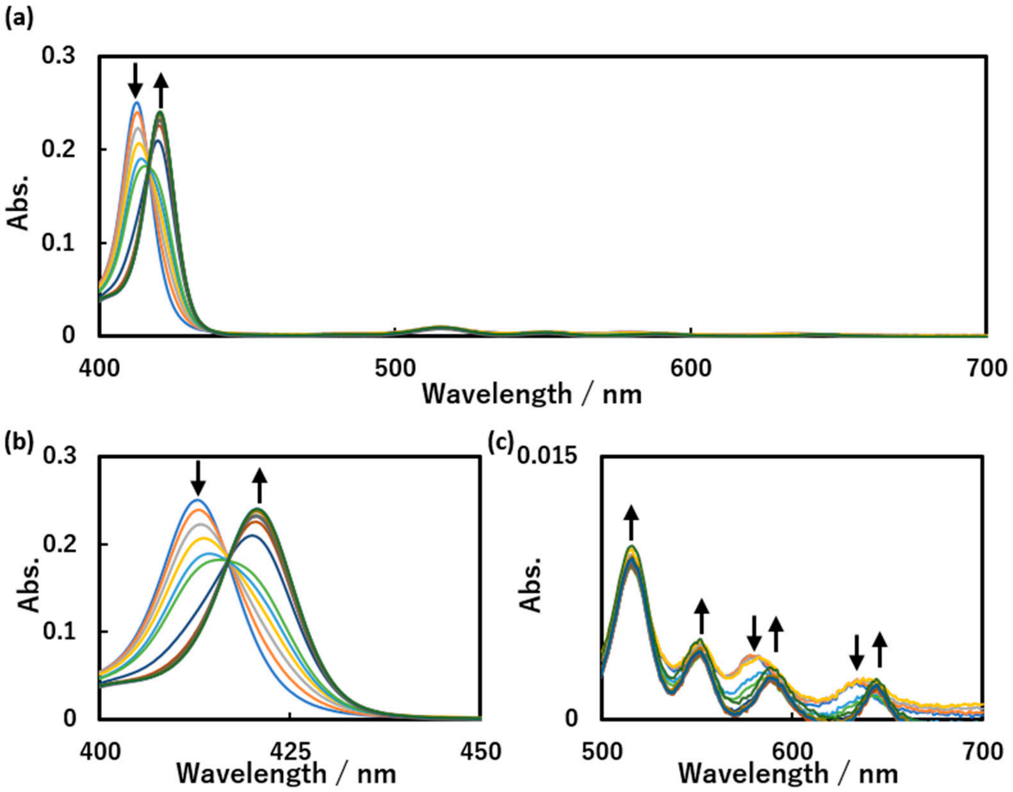

Figure S6. (a) Absorption spectra of 0.6 μM TPPS upon the addition of 0, 2.4 μM, 4.8 μM, 7.2 μM, 9.6 μM, 12.0 μM, 24.0 μM, 36.0 μM, 48.0 μM, 72.0 μM, 0.3 mM, and 2.9 mM PVP in 0.01 M phosphate buffer (pH 8.0); (b) zoomed spectra of 400–500 nm of Figure S6a; (c) zoomed spectra of 500–700 nm of Figure S6a.

Absorption spectra were acquired for mixed solutions of TPPS (12μM) and PVP (0, 0.05 mM, 0.10 mM, 0.14 mM, 0.19 mM, 0.24 mM, 0.48 mM, 0.72 mM, 0.96 mM, 1.44 mM, 5.76 mM, and 0.06 M) in 0.01 M phosphate buffer (pH 8.0) (Figure S7). Redshifts in the regions of the Soret band and the Q-bands were observed. The accuracy of the Soret band is not good due to the high concentration of ZnTPPS.

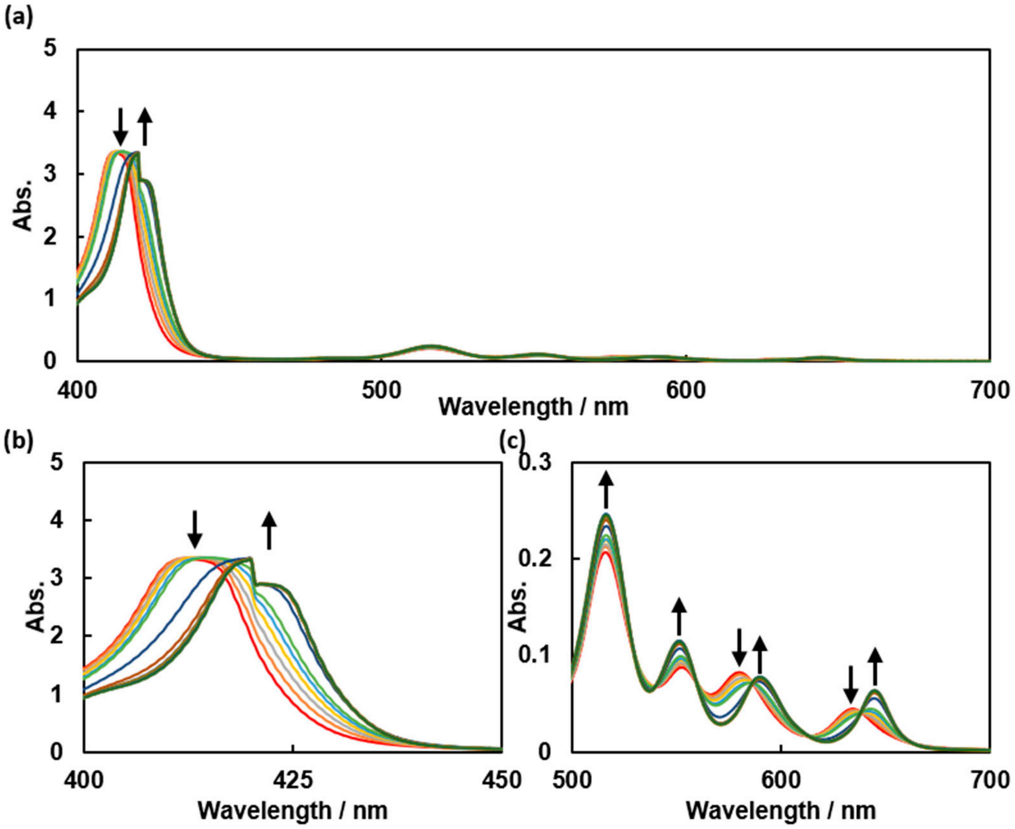

Figure S7. (a) Absorption spectra of 12 μM TPPS upon the addition of 0, 0.05 mM, 0.10 mM, 0.14 mM, 0.19 mM, 0.24 mM, 0.48 mM, 0.72 mM, 0.96 mM, 1.44 mM, 5.76 mM, and 0.06 M PVP in 0.01 M phosphate buffer (pH 8.0); (b) zoomed spectra of 400–500 nm of Figure S7a; (c) zoomed spectra of 500–700 nm of Figure S7a.

Absorption spectra of TPPS upon the addition of PVP in 0.01 M phosphate buffer (pH 4.0)

Absorption spectra were acquired for mixed solutions of TPPS (0.6 μM) and PVP (0, 2.4 μM, 4.8 μM, 7.2 μM, 9.6 μM, 12.0 μM, 24.0 μM, 36.0 μM, 48.0 μM, 72.0 μM, 0.3 mM, and 2.9 mM) in 0.01 M phosphate buffer (pH 4.0) (Figure S8). Shifts in the region of the Soret band and changing in the absorbance intensity of the Q-bands were observed. The accuracy of the Q-bands was not good due to the low concentration of TPPS. So, absorption spectra were detected again in 20 folds the concentrations of TPPS and PVP.

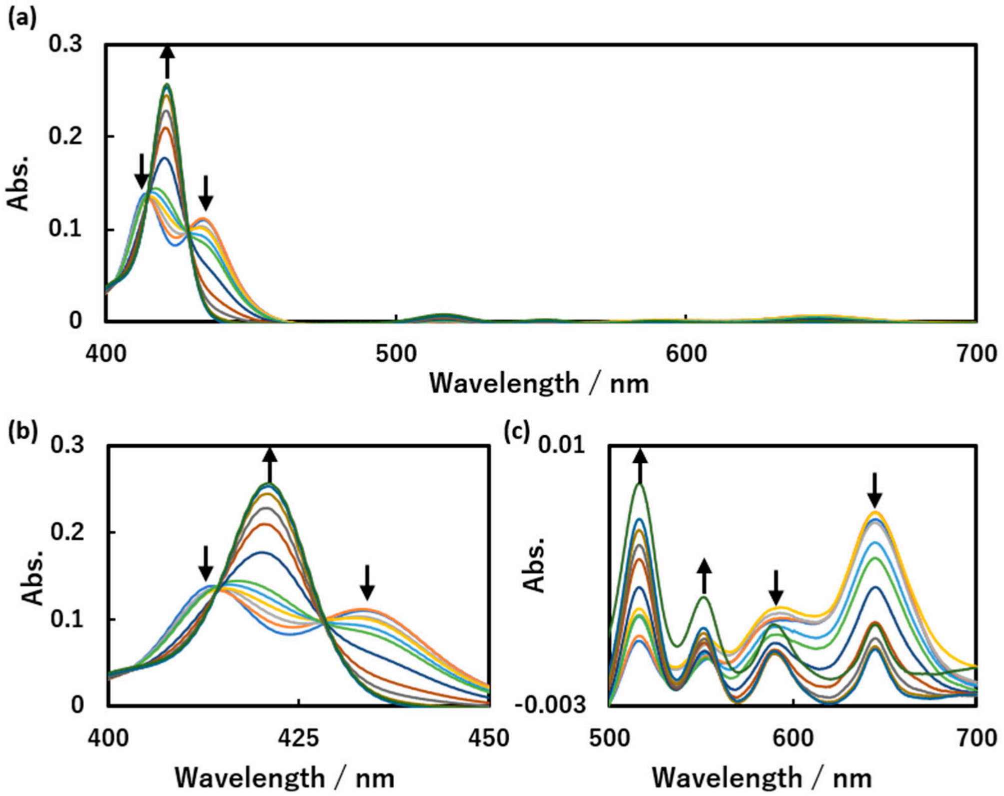

Figure S8. (a) Absorption spectra of 0.6 μM TPPS upon the addition of 0, 2.4 μM, 4.8 μM, 7.2 μM, 9.6 μM, 12.0 μM, 24.0 μM, 36.0 μM, 48.0 μM, 72.0 μM, 0.3 mM, and 2.9 mM PVP in 0.01 M phosphate buffer (pH 4.0); (b) zoomed spectra of 400–500 nm of Figure S8a; (c) zoomed spectra of 500–700 nm of Figure S8a.

Absorption spectra were acquired for mixed solutions of TPPS (12 μM) and PVP (0, 0.05 mM, 0.10 mM, 0.14 mM, 0.19 mM, 0.24 mM, 0.48 mM, 0.72 mM, 0.96 mM, 1.44 mM, 5.76 mM, and 0.06 M) in 0.01 M phosphate buffer (pH 4.0) (Figure S9). Shifts in the region of the Soret band and changing in the absorbance intensity of the Q-bands were observed. The accuracy of the Soret band is not good due to the high concentration of ZnTPPS.

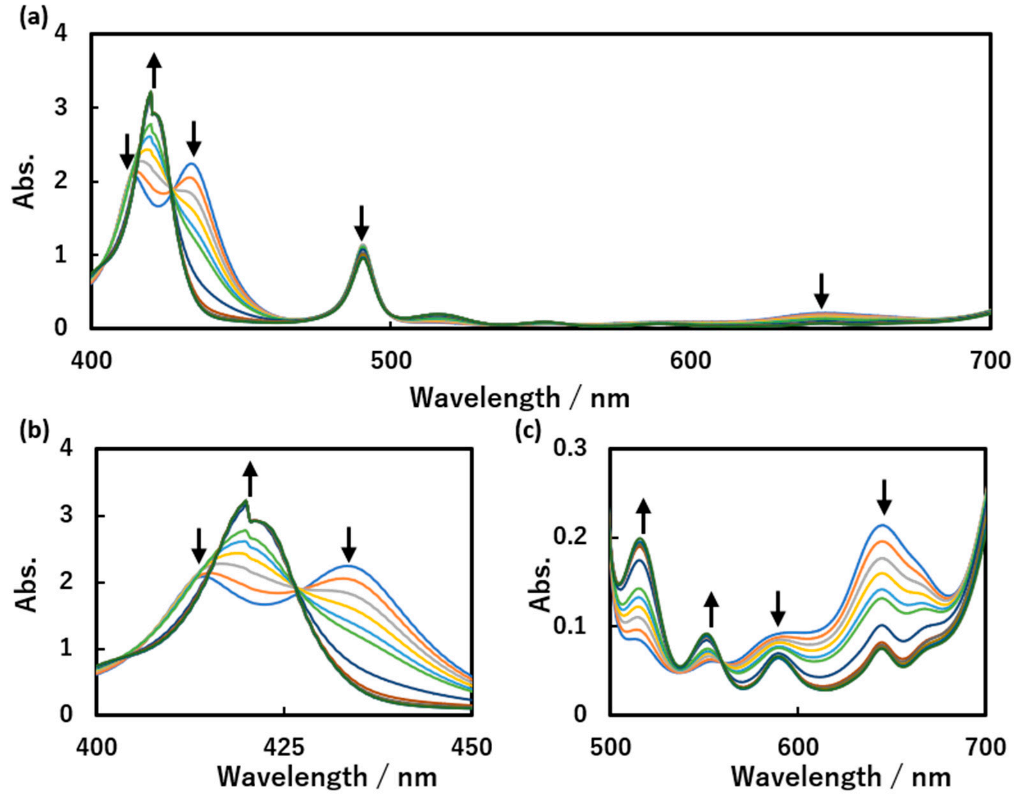

Figure S9. (a) Absorption spectra of 12 μM TPPS upon the addition of 0, 0.05 mM, 0.10 mM, 0.14 mM, 0.19 mM, 0.24 mM, 0.48 mM, 0.72 mM, 0.96 mM, 1.44 mM, 5.76 mM, and 0.06 M PVP in 0.01 M phosphate buffer (pH 4.0); (b) zoomed spectra of 400–500 nm of Figure S9a; (c) zoomed spectra of 500–700 nm of Figure S9a.

Fluorescence spectra of ZnTPPS and TPPS in the absence and presence of PVP

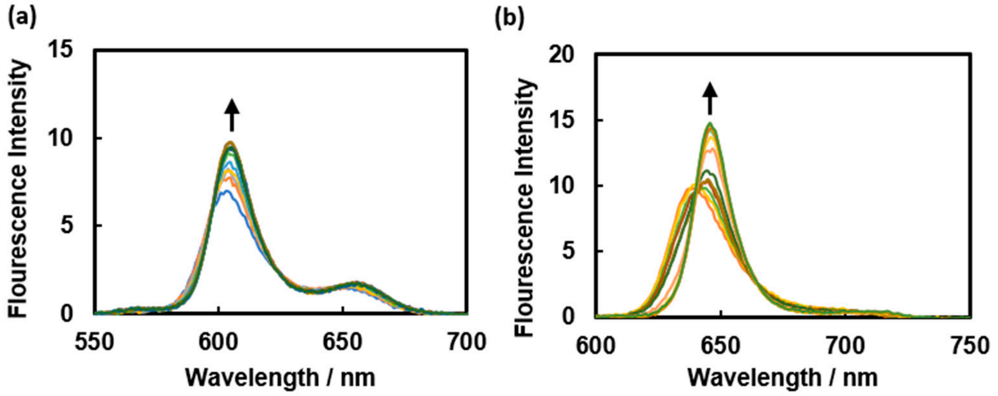

Figure S10. (a) Fluorescence spectra of 0.6 μM ZnTPPS upon the addition of 0, 2.4 μM, 4.8 μM, 7.2 μM, 9.6 μM, 12.0 μM, 24.0 μM, 36.0 μM, 48.0 μM, 72.0 μM, 0.3 mM, and 2.9 mM PVP in 0.01 M phosphate buffer (pH 8.0); (b) Fluorescence spectra of 0.6 μM TPPS upon the addition of 0, 2.4

μM, 4.8 μM, 7.2 μM, 9.6 μM, 12.0 μM, 24.0 μM, 36.0 μM, 48.0 μM, 72.0 μM, 0.3 mM, and 2.9 mM PVP in 0.01 M phosphate buffer (pH 8.0).

Fluorescence spectra of mixed solutions of ZnTPPS or TPPS (0.6 μM) and PVP (0–2.9 mM, 0–4,800 equiv.) in 0.01 M phosphate buffer (pH 8.0) were acquired (Figure S10). Redshifts and an enhancement of the intensity were observed upon the addition of PVP, suggesting that the PVP stabilized the porphyrins.

Absorption spectra of ZnTPPS and TPPS in the absence and presence of *N*-methyl-2-pyrrolidone (NMP) or *N*-vinyl-2-pyrrolidone (NVP)

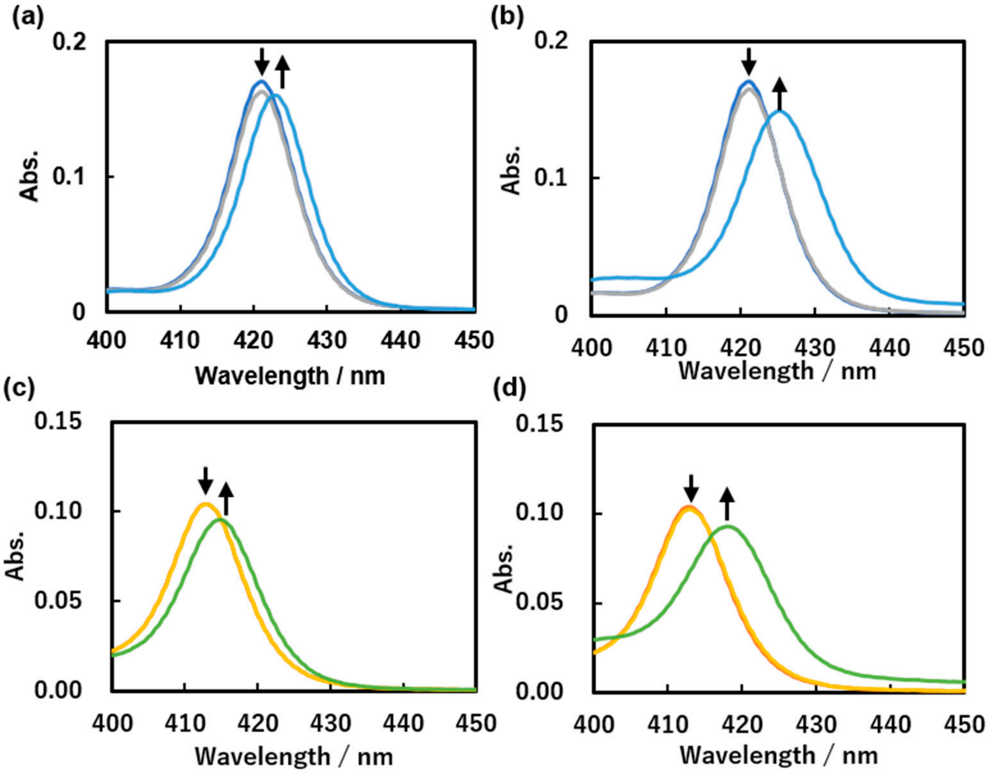

Figure S11. (a) Absorption spectra of 0.6 μM ZnTPPS in the absence and presence of 0, 2.9 mM and 0.3 M NMP; (b) absorption spectra of 0.6 μM TPPS in the absence and presence of 0, 2.9 mM and 0.3 M NVP; (c) absorption spectra of 0.6 μM ZnTPPS in the absence and presence of 0, 2.9 mM and 0.3 M NMP; (d) absorption spectra of 0.6 μM TPPS in the absence and presence of 0, 2.9 mM and 0.3 M NVP.

Absorption spectra were recorded for mixed solutions of ZnTPPS or TPPS (0.6 μM) and NMP (0–0.3 M, 0–480,000 equiv.) or NVP (0–0.3 M, 0–480,000 equiv.) in 0.01 M phosphate buffer (pH 8.0). Soret bands in the spectra of both ZnTPPS and TPPS were slightly shifted upon the addition of NMP or NVP (Figure S11), whereas the Soret bands dramatically shifted when PVP was added

in the same amount as NMP. These results suggest that the polymer structure of PVP played a critical role in the formation of complexes with ZnTPPS and TPPS.

Absorption spectra of ZnTPPS in the absence and presence of PEG or PVP with different molecular weights

Absorption spectra were acquired for solution of ZnTPPS (0.6 μM) and mixed solutions of ZnTPPS (0.6 μM) and PEG 6000 (Mn = 6,000, 24.0 mM) or PVP (PVP 25 (Mη = 24,500), PVP K-30 (Mη = 40,000), and PVP K-90 (Mη = 360,000), 24.0 mM) in 0.01 M phosphate buffer (pH 8.0) (Figure S12).

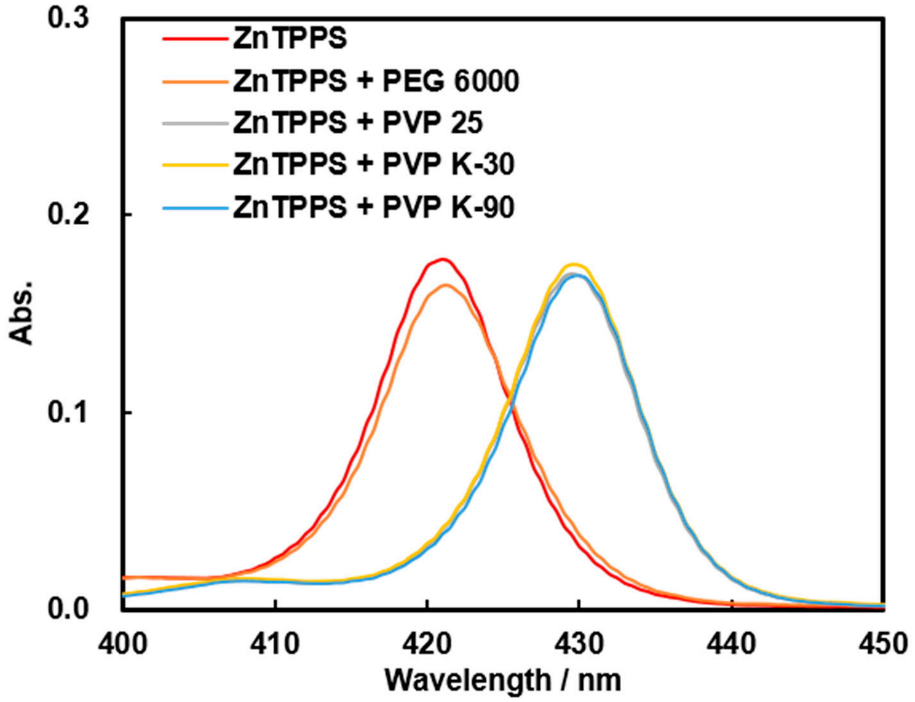

Figure S12. Absorption spectra of 0.6 μM ZnTPPS in the absence and presence of PEG or PVP.

In the presence of three kinds of PVP, Soret band of ZnTPPS shifted large-ranged to 430 nm, while only a 1 nm redshift was observed in the presence of PEG, which suggests that there are much weaker (or no) interactions between ZnTPPS and PEG than that of ZnTPPS and PVP, and similar interactions between ZnTPPS and three kinds of PVP.

Fluorescence quenching of ZnTPPS and TPPS by MV^{2+} in the absence and presence of PVP

Fluorescence quenching of ZnTPPS or TPPS (0.6 μM) by MV^{2+} (0–0.44 mM) in 0.01 M phosphate buffer (pH 8.0) was observed (Figures S13 and S14). Quenching was reduced in the presence of PVP (24.0 μM), suggesting a more burdensome electron-transfer environment.

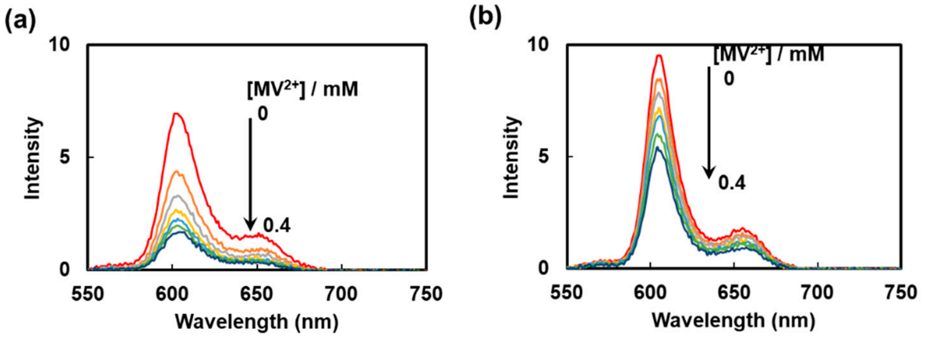

Figure S13. (a) Fluorescence spectra of 0.6 μM ZnTPPS in the absence of PVP with MV^{2+} added in 0.07 mM intervals up to 0.44 mM; (b) Fluorescence spectra of 0.6 μM ZnTPPS in the presence of PVP (24.0 μM) with MV^{2+} added in 0.07 mM intervals up to 0.44 mM.

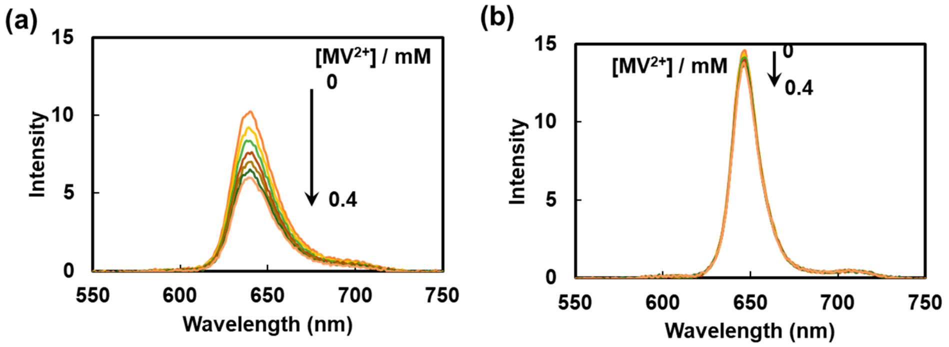

Figure S14. (a) Fluorescence spectra of 0.6 μM TPPS in the absence of PVP with MV^{2+} added in 0.07 mM intervals up to 0.44 mM; (b) Fluorescence spectra of 0.6 μM TPPS in the presence of PVP (2.4×10^{-5} M) with MV^{2+} added in 0.07 mM intervals up to 0.44 mM.

Fluorescence quenching of ZnTPPS by MV^{2+} in the presence of PVP and absence of oxygen

Fluorescence quenching of ZnTPPS (0.6 μM) by MV^{2+} (0–0.44 mM) in 0.01 M phosphate buffer (pH 8.0) was observed in the presence of PVP and absence of oxygen (Figures S15a). Referring to Figure S15a, Stern–Volmer (SV) plots for the quenching of the emission of the ZnTPPS were constructed (Figure S15b).

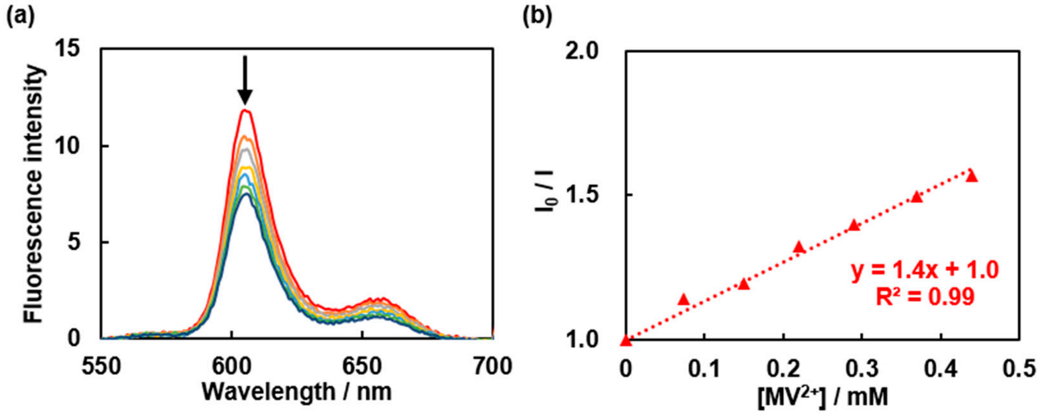

Figure S15. (a) Fluorescence spectra of 0.6 μM ZnTPPS in the presence of PVP (24.0 μM) with MV^{2+} added in 0.07 mM intervals up to 0.44 mM in the absence of oxygen; (b) Stern–Volmer plots of the quenching of ZnTPPS by MV^{2+} in the absence of oxygen.

Absorption spectra of ZnTPPS upon the successive addition of MV^{2+}in the presence of PEG or PVP with different molecular weights

Absorption spectra of ZnTPPS (0.6 μM) upon the successive addition of MV^{2+} (0–0.44 mM) in the presence of PEG or PVP were recorded in 0.01 M phosphate buffer (pH 8.0) (Figure S15).

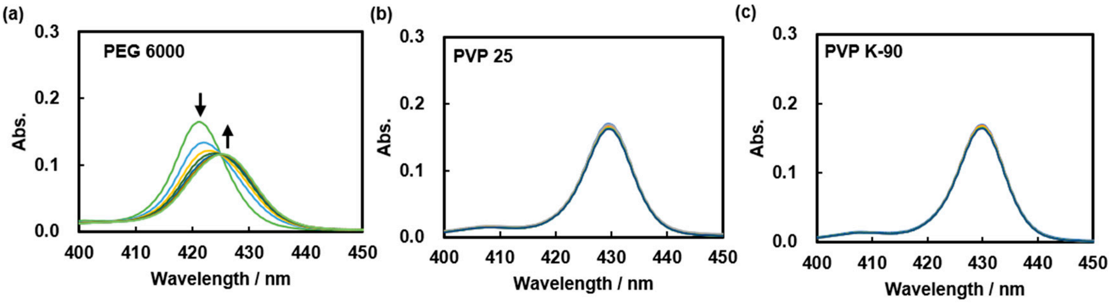

Figure S16. (a) Absorption spectra of 0.6 μM ZnTPPS with MV^{2+} added in 0.07 mM intervals up to 0.44 mM in the presence of PEG; (b) Absorption spectra of 0.6 μM ZnTPPS with MV^{2+} added in 0.07 mM intervals up to 0.44 mM in the presence of PVP 25; (c)Absorption spectra of 0.6 μM ZnTPPS with MV^{2+} added in 0.07 mM intervals up to 0.44 mM in the presence of PVP K-90.

Redshifts were observed in the presence of PEG, while negligible peak shifts were observed in the presence of PVP, suggesting that PEG cannot affect the interaction between ZnTPPS and MV^{2+}, and the restraining of interaction between ZnTPPS and MV^{2+} has no PVP molecular weight dependence.

Fluorescence quenching of ZnTPPS by MV^{2+} in the presence of PEG or PVP with different molecular weights

Fluorescence quenching of ZnTPPS (0.6 μM) by MV^{2+} (0–0.44 mM) in the presence of 0.24 mM PEG or PVP in 0.01 M phosphate buffer (pH 8.0) was observed (Figure S16).

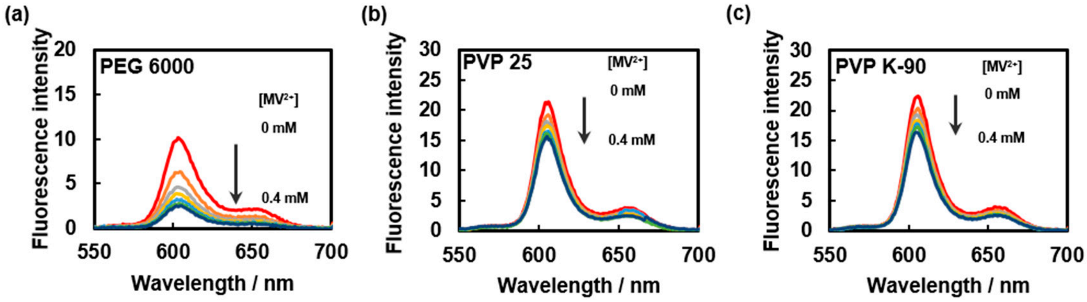

Figure S17. (a) Fluorescence spectra of 0.6 μM M ZnTPPS upon the successive addition of 0–0.44 mM MV^{2+} in the presence of 0.24 mM PEG; (b) Fluorescence spectra of 0.6 μM ZnTPPS upon the successive addition of 0–0.44 mM MV^{2+} in the presence of 0.24 mM PVP 25; (c) Fluorescence spectra of 0.6 μM ZnTPPS upon the successive addition of 0–0.44 mM MV^{2+} in the presence of 0.24 mM PVP K-90.

Stern-Volmer plots were made according to Figures S13 and S16 (Figure S17). PEG has little effect on the photoinduced electron transfer between ZnTPPS and MV^{2+}, and the controlling of photoinduced electron transfer by PVP showed no PVP molecular weight dependence.

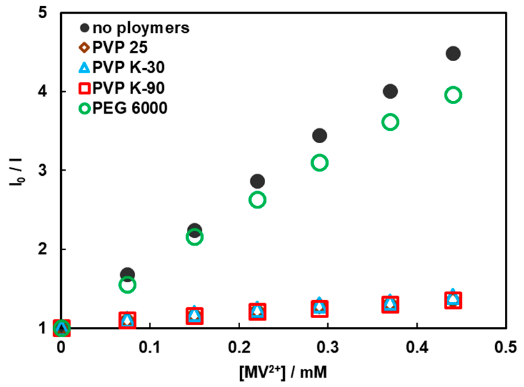

Figure S18. Stern–Volmer plots of the quenching of ZnTPPS by MV^{2+} in the absence (solid circles) and presence of 0.24 mM PEG (hollow circles) or PVP (25 (hollow diamonds), K-30 (hollow triangles), and K-90 (hollow squares)).

Generation of the electron transfer product $MV^{+\bullet}$

Mixed solution 10 mL containing ZnTPPS (6.0 μM), PVP (0, 24.0 μM, 240.0 μM, 2.4 mM and 24.0 mM), MV^{2+} (1.0 mM) and EDTA-4Na (10.0 mM) was degassed by 15 min N_2 bubbling. Then, the solution was transferred to three 3mL cuvettes using a syringe and irradiated by UV light at ~20 cm for 30 min. The generation of the electron-transfer product $MV^{+\bullet}$ after 30 min of irradiation by UV light was observed (Figure S18). Based on our experience, absorbance at 605 nm and concentration of $MV^{+\bullet}$ have a linear relationship in our experimental conditions, so absorbance at 605 nm was used to modulate the generated amounts of $MV^{+\bullet}$. With increasing PVP concentration, the generated amount of $MV^{+\bullet}$ increased and tended to become saturated at high PVP concentrations.

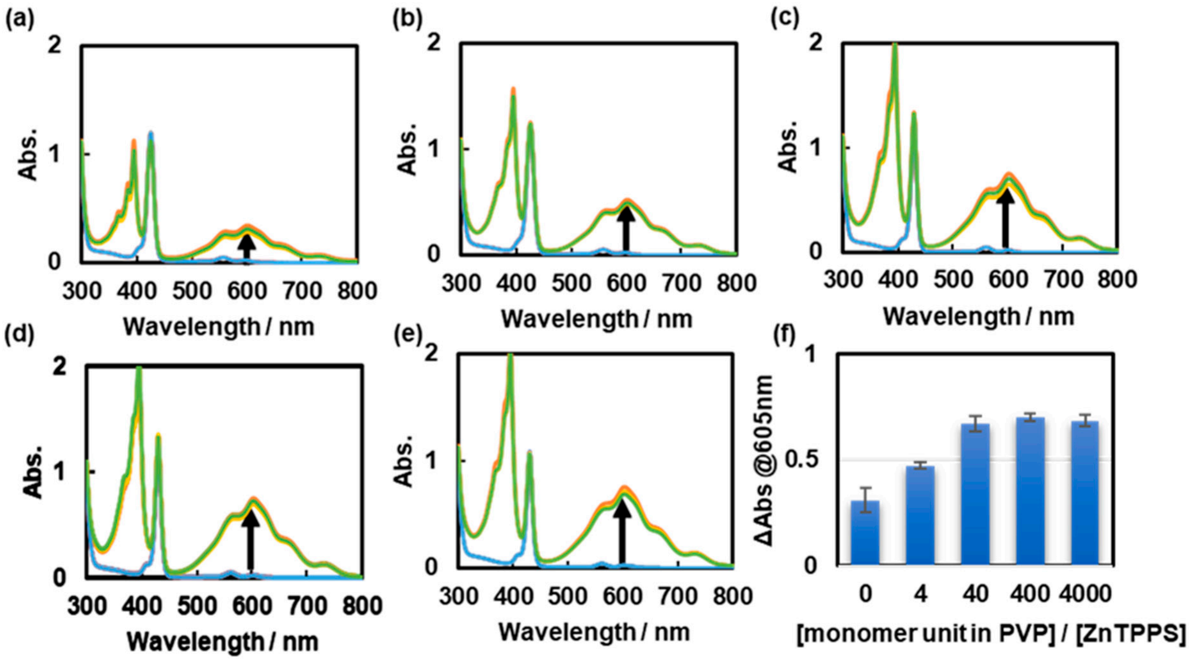

Figure S19. (a) Absorption spectra of mixed solutions of 6.0 μM ZnTPPS, 1.0 mM MV^{2+}, and 10.0 mM EDTA in the absence PVP before and after 30 min of irradiation under UV light; (b) absorption spectra of mixed solutions of 0.6 μM ZnTPPS, 1.0 mM MV^{2+}, and 10.0 mM EDTA in the presence of 24.0 μM PVP before and after 30 min of irradiation under UV light; (c) absorption spectra of mixed solutions of 6.0 μM ZnTPPS, 1.0 mM MV^{2+}, and 10.0 mM EDTA in the presence of 0.24 mM PVP before and after 30 min of irradiation under UV light; (d) absorption spectra of mixed solutions of 6.0 μM ZnTPPS, 1.0 mM MV^{2+}, and 10.0 mM EDTA in the presence of 2.4 mM PVP before and after 30 min of irradiation under UV light; (e) absorption spectra of mixed solutions of 6.0 μM ZnTPPS, 1.0 mM MV^{2+}, and 10.0 mM EDTA in the presence of 24.0 mM PVP before and after 30 min of irradiation under UV light; (f) change of the absorption at 605 nm as a function of the [monomer unit in PVP]/[ZnTPPS].

Generation of the electron transfer product $MV^{+\bullet}$ in the presence of PEG or PVP with different molecular weights

The generation of the electron-transfer product $MV^{+\bullet}$ after 30 min of irradiation by UV light was observed in the presence of PEG or PVP (25, K-90) (Figure S19).

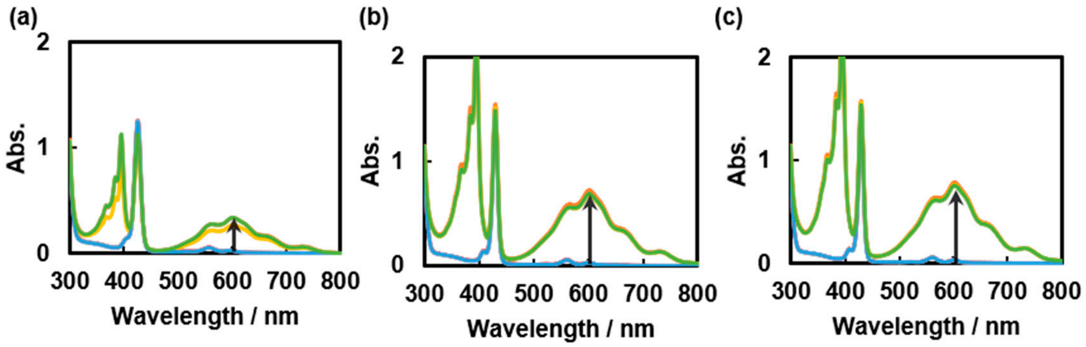

Figure S20. (a) Absorption spectra of mixed solutions of 6.0 μM ZnTPPS, 1.0 mM MV^{2+}, and 10.0 mM EDTA in the presence of 0.24 mM PEG before and after 30 min of irradiation under UV light; (b) absorption spectra of mixed solutions of 6.0 μM ZnTPPS, 1.0 mM MV^{2+}, and 10.0 mM EDTA in the presence of 0.24 mM PVP 25 before and after 30 min of irradiation under UV light; (c) absorption spectra of mixed solutions of 6.0 μM ZnTPPS, 1.0 mM MV^{2+}, and 10.0 mM EDTA in the presence of and 0.24 mM PVP K-90 before and after 30 min of irradiation under UV light.

The absorption at 605 nm corresponding to $MV^{+\bullet}$ was used to modulate the generated amounts of $MV^{+\bullet}$ (Figure S20).

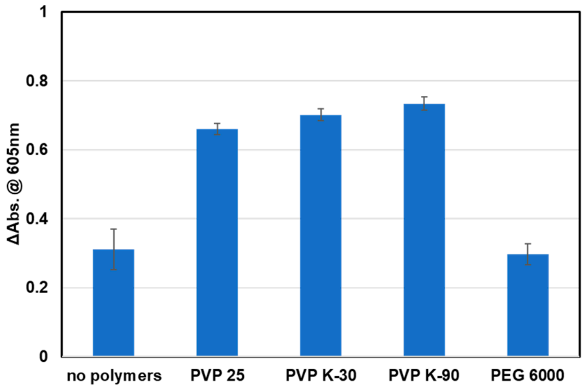

Figure S21. Change of the absorption at 605 nm as a function of the added polymers.

More $MV^{+\bullet}$ generated in the presence of PVP, and when increasing the molecular weight of PVP, the generated amount of $MV^{+\bullet}$ increased, while the same or even less amount of $MV^{+\bullet}$ generated in the presence of PEG. PVP plays a critical role in this photoinduced electron-transfer process.

References

1. Flamigni, L.; Talarico, A. M.; Ventura, B.; Rein, R.; Solladie, N. A versatile bis-porphyrin tweezer host for the assembly of noncovalent photoactive architectures: A photophysical characterization of the tweezers and their association with porphyrins and other guests. *Chem. Eur. J.* **2006**, *12*, 701-712.

2. Nappa, M.; Valentine, J. S. The influence of axial ligands on metalloporphyrin visible absorption spectra. Complexes of tetraphenylporphinatozinc. *J. Am. Chem. Soc.* **1978**, *100*, 5075-5080.
